# LEDGF/p75 Deficiency Increases Deletions at the HIV-1 cDNA Ends

**DOI:** 10.3390/v9090259

**Published:** 2017-09-15

**Authors:** Murilo T. D. Bueno, Daniel Reyes, Manuel Llano

**Affiliations:** Department of Biological Sciences, University of Texas at El Paso. El Paso, TX 79968, USA; murilotdb@outlook.com (M.T.D.B.); dsr8721@gmail.com (D.R.)

**Keywords:** LEDGF/p75, HIV-1, 2-LTR circles, unintegrated HIV-1 cDNA

## Abstract

Processing of unintegrated linear HIV-1 cDNA by the host DNA repair system results in its degradation and/or circularization. As a consequence, deficient viral cDNA integration generally leads to an increase in the levels of HIV-1 cDNA circles containing one or two long terminal repeats (LTRs). Intriguingly, impaired HIV-1 integration in LEDGF/p75-deficient cells does not result in a correspondent increase in viral cDNA circles. We postulate that increased degradation of unintegrated linear viral cDNA in cells lacking the lens epithelium-derived growth factor (LEDGF/p75) account for this inconsistency. To evaluate this hypothesis, we characterized the nucleotide sequence spanning 2-LTR junctions isolated from LEDGF/p75-deficient and control cells. LEDGF/p75 deficiency resulted in a significant increase in the frequency of 2-LTRs harboring large deletions. Of note, these deletions were dependent on the 3′ processing activity of integrase and were not originated by aberrant reverse transcription. Our findings suggest a novel role of LEDGF/p75 in protecting the unintegrated 3′ processed linear HIV-1 cDNA from exonucleolytic degradation.

## 1. Introduction

Linear HIV-1 cDNA is generated by reverse transcription of the HIV-1 RNA genome and rendered integration-competent by integrase. The latter viral enzyme recognizes an imperfect inverted repeat structure formed by the terminal 10 to 12 nucleotides of the HIV-1 cDNA denominated att and removes the 2 terminal nucleotides from each 3′ end. The resulting 3′ processed viral cDNA is then integrated into the host genome by the strand-transfer activity of integrase with the contribution of the chromatin-bound protein lens epithelium-derived growth factor (LEDGF/p75) that tethers to chromatin integrase associated with HIV-1 cDNA in a pre-integration complex.

Upon nuclear import, the linear form of the HIV-1 cDNA is targeted for degradation or circularization by the host DNA repair system. As a result, depletion of enzymes implicated in the degradation pathway leads to an increase in retroviral transduction and formation of provirus and 2-LTR circles [1]. Several host DNA nucleotide excision repair proteins such as DNA helicases xeroderma pigmentosum group B and D (XPB and XPD, respectively) [2,3], and Uracil DNA glycosylase 2 [4] and the component of the homologous recombination repair system Rad52 [5] have been implicated in this degradation process. Therefore, the HIV-1 cDNA degradation pathway acts as an anti-viral mechanism that targets HIV-1 at a pre-integration step earlier than circularization. In contrast, depletion of proteins implicated in circularization, that involves the joining of the 5′ and 3′ ends of linear HIV-1 cDNA by the non-homologous DNA end joining (NHEJ) pathway [6,7,8], is associated with a decrease in HIV-1 infection, formation of proviruses and circularized HIV-1 cDNA harboring two long terminal repeats (2-LTRs) [7,8].

Furthermore, HIV-1 cDNA circularization contributes to eliminate linear forms of HIV-1 cDNA that fail to integrate. Pharmacological or mutation-driven inhibition of the catalytic activity of HIV-1 integrase results in decreased viral cDNA integration and significant increase in the amount of circularized HIV-1 cDNA [9,10,11,12,13,14,15]. Unexpectedly, while LEDGF/p75 deficiency drastically reduces HIV-1 cDNA integration by 8–10 folds, the levels of 2-LTR circles are only modestly increased by less than two-fold [15,16,17,18], despite a negligible role of LEDGF/p75 in 2-LTR circle formation [15]. Similarly to LEDGF/p75 depletion, overexpression of DNA repair enzymes mediating HIV-1 cDNA degradation decreases retroviral integration without a concomitant increase in the levels of 2-LTR circles [2,3,4,5]. Therefore, we postulate that in the absence of LEDGF/p75, the levels of 2-LTRs do not significantly increase despite the severe defect in integration because of increased degradation of unintegrated linear HIV-1 cDNA. In further support of this mechanism, it was reported that blockage of integration by the integrase strand transfer inhibitor Raltegravir caused a significantly greater increase in 2-LTR circles in control than in LEDGF/p75-deficient cells [15].

To evaluate the role of LEDGF/p75 in the degradation of the HIV-1 cDNA, we performed a detailed sequence analysis of 2-LTR circle junctions isolated from infected LEDGF/p75-deficient and control cells. The DNA sequence at the 2-LTR junctions is thought to be identical to the viral cDNA ends prior to circularization. Therefore, the analysis of 2-LTR junctions is a simple and valuable experimental tool that can be exploited to determine the identity of the ends of the linear viral cDNA molecules which failed to integrate into the host genome.

Our results indicate that LEDGF/p75 deficiency is associated with a significantly higher frequency of deletions at 2-LTR junctions. The incidence of large deletions (more than 10 nucleotides) was particularly favored in the absence of LEDGF/p75. Importantly, integrase-mediated 3′ processing was required for the generation of these deletions. Furthermore, nucleotide deletions at the 2-LTR junctions were not originated by abnormal RT activity. Taken together, our findings indicate that LEDGF/p75 protects the HIV-1 cDNA from degradation providing a mechanism for the unexpected low levels of 2-LTR circles found in HIV-1 infected LEDGF/p75-deficient cells and suggesting a novel role of LEDGF/p75 in HIV-1 cDNA integration.

## 2. Materials and Methods

### 2.1. LEDGF/p75-Deficient Cell Lines

Cells derived from the human CD4+ T cell line SupT1 were used in this study. LEDGF/p75-deficient (T_L3_) and control (T_C3_) cells were generated by transduction of SupT1 cells with HIV-derived vectors expressing a LEDGF/p75-specific or a scrambled shRNA, respectively [16]. This strategy resulted in 97% downregulation of the LEDGF/p75 mRNA levels in T_L3_ as compared to T_C3_ cells [16]. SupT1-derived cell lines were grown in RPMI1640 supplemented with 10% of heat-inactivated fetal calf serum, 2 mM l-glutamine and 1% penicillin/streptomycin.

### 2.2. Generation of Retroviral Vectors

HIVluc, a single-round reporter virus that carries a deletion in the *env* gene and the gene for firefly luciferase in *nef* [16] and HIV-1_D64N_ (a gift from Alan Engelman) were produced in HEK 293T cells by calcium-phosphate co-transfection of 15 µg of the corresponding expression plasmids and 5 µg of the Vesicular Stomatitis Virus glycoprotein G (VSV-G) expression plasmid, pMD.G (a gift from Didier Trono). Then, 48 h after transfection, the viral supernatants were harvested and concentrated by ultracentrifugation at 124,750× *g* for 2 h on a 20% sucrose cushion. Potential traces of the HIV-1 expression plasmid used during viral production were removed by treating 1 ml of concentrated viral supernatant with 100 µL of turbo DNase (Thermo Fisher Scientific, Waltham, MA, USA) for 45 min at 37 °C. HEK293T cells were grown in Dulbecco’s Modified Eagle’s medium (DMEM) supplemented with 10% of heat-inactivated fetal calf serum, 2 mM/l-glutamine and 1% penicillin/streptomycin.

### 2.3. Single-Round Viral Infectivity Assay

T_L3_ and T_C3_ cells were plated at 10^5^ cells in 500 µL of RPMI1640 culture medium in 24-well plates and infected with HIVluc viral supernatant. Cells were collected 5 days post-infection by centrifugation at 1000× *g* for 6 min and the pellet lysed in 100 µL of PBS-1% Tween 20 for 15 min on ice. Cellular lysates were centrifuged at 22,000× *g* for 2 min and supernatant was used for quantification of luciferase activity. An aliquot of 20 µL of the cellular lysate supernatant was mixed with 45 µL of substrate (Bright-Glow™ Luciferase Assay System, Promega, Fitchburg, WI, USA) and luciferase activity was quantified using a microplate luminometer.

### 2.4. Analysis of HIV-1 2-LTR Circles

Cellular DNA extracted from T_C3_ and T_L3_ cells from 4 independent infection experiments performed in different days and with different HIV-1 preparations was pooled and used for HIV-1 2-LTR circles analysis. DNA was extracted 24 h post-infection (High pure PCR template preparation kit, Roche, Penzberg, Germany) and 2-LTR junctions were amplified by PCR using Phusion™ High-Fidelity DNA Polymerase (New England Biolabs, Ipswich, MA, USA). Amplification was performed in a MiniOpticon system (Bio-rad, Hercules, CA, USA) using 500 ng of total DNA and primers MB5 5′-TATAGCGGCCGCAACTAGGGAACCCACTGCTTAAG-3′ and MB6 5′-TATATCTAGAATCCACAGATCAAGGATATCTTGTC-3′ at 20 pmol per reaction. Annealing was performed at 52 °C and extension at 72 °C both for 30 s, 40 cycles were used. Optimal annealing temperature was established experimentally by PCR analysis using as template a plasmid containing 2-LTRs in the presence of uninfected cell genomic DNA. The 204 base pairs PCR product obtained from the infected cells was resolved in a 1% agarose gel and DNA was isolated from a gel fragment spanning the region from 100 to 300 base pairs. Purified amplified DNA was cloned into *Not* I/*Xba* I in pCDNA 3.1 (+). Positive colonies were identified by *Hind* III restriction analysis and sequenced using the MB5 primer. To reach a significant number of unique 2-LTR junctions multiple PCR/molecular cloning rounds were performed and only clones containing unique junction sequences were considered for analysis.

### 2.5. HIV-1 cDNA Analysis

DNA extracted 24 h after HIVluc infection was also used to quantify total HIV cDNA (gag DNA) and 2-LTR circles by real time PCR. Amplifications were performed in a MiniOpticon system (Bio-rad, Hercules, CA, USA) using 20 ng of total DNA with primers and conditions previously described [16]. Levels of 2-LTR and total HIV-1 (gag) cDNA were normalized for total HIV-1 (gag) and mitochondrial DNA, respectively, to guarantee equal loading. Mitochondrial DNA was determined using primers and procedures previously described [16]. Fold change was calculated using the Δ*C*t method as recommended in the thermo-cycler manual and differences were expressed considering the levels found in T_C3_ cells as 1.

### 2.6. Immunoblotting

10^6^ T_C3_ and T_L3_ cells were directly lysed in 2× Laemmli sample buffer (4% sodium dodecyl sulfate, 20% Glycerol, 0.02% bromophenol blue, 120 mM Tris-Cl pH 6.8) and the cellular lysates were resolved by SDS-PAGE and transferred overnight to a Polyvinylidene (PDVF) membrane at 100 mA at 4 °C. Membrane was blocked in Tris-buffered saline (TBS) (25 mM Tris HCl, 150 mM NaCl, pH 7.6) containing 10% milk for 1 h and then incubated overnight at 4 °C with anti-LEDGF mAb (611714 BD Transduction Laboratories. 1/500) in TBS-5% milk-0.05% Tween 20 (antibody dilution buffer). As loading control, α tubulin was detected with a specific mAb (clone B-5-1-2, Sigma-Aldrich, St. Louis, MO, USA) at a 1/4000 dilution for 2 h at 25 °C. Membrane was washed in TBS-0.1% Tween 20 and bound antibodies detected with goat anti-mouse Igs-HRP (Sigma-Aldrich, St. Louis, MO, USA) diluted 1/2000 in antibody dilution buffer followed by chemoluminescence detection as described before [19].

### 2.7. Statistical Analysis

Statistical significance of the observed experimental differences was determined using the *Z*-test for 2 proportions with confidence interval of 95%, as described in [20].

## 3. Results

### 3.1. Susceptibility of T_L3_ and T_C3_ Cells to HIV-1 Infection

**Figure 1:**
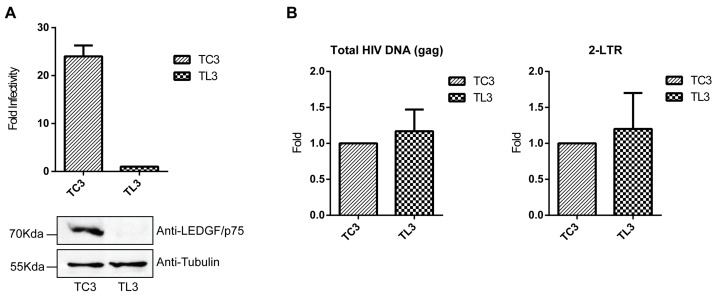
HIV-1 infection of control (T_C3_) and lens epithelium-derived growth factor (LEDGF/p75)-deficient (T_L3_) cells. (**A**) Single-round infection of T_C3_ and T_L3_ cells. Cells were challenged with HIVluc and luciferase activity was analyzed 5 days post-infection. Luciferase levels detected in T_L3_ cells were used for normalization. Bars represent the mean and standard deviation values calculated from 4 independent infection experiments performed on different days with different viral preparations. Levels of LEDGF/p75 were determined by immunoblotting with a specific antibody against LEDGF/p75. Equal protein loading was accessed by immunoblotting with anti-Tubulin; (**B**) real time PCR quantification of total HIV cDNA (gag DNA) and circularized HIV-1 cDNA containing two long terminal repeats (2-LTRs) isolated from HIVluc-infected T_C3_ and T_L3_ cells. Bars represent the mean and standard deviation of values calculated from 2 independent infection experiments. Levels found in T_C3_ cells were used for normalization.

SupT1 LEDGF/p75-deficient (T_L3_) and control cells (T_C3_) (Figure 1A, lower panel) were infected with HIVluc and analyzed 5 days post-infection for luciferase activity. T_L3_ cells were severely resistant to HIV-1 infection (Figure 1A, upper panel) due to the defect in viral DNA integration previously reported in these cells [16,19,21].

The levels of total HIV-1 cDNA (gag DNA) and 2-LTR circles were quantified using DNA isolated from these cells 24 h post-infection with HIVluc. As previously reported [15,16,17,18], similar levels of total HIV cDNA (gag DNA) were found in LEDGF/p75-deficient and control cells (Figure 1B). However, the levels of 2-LTR circles, expected to be elevated in LEDGF/p75-deficient cells as a consequence of a defect in HIV-1 integration, were nearly equivalent in T_L3_ and T_C3_ cells (Figure 1B). Similar results indicating only a modest increase (approximately 2-fold) in levels of 2-LTR circles in LEDGF/p75-deficient cells were previously reported [15,16,17,18]. Thus, LEDGF/p75 depletion significantly decreases HIV-1 cDNA integration but does not promote a reciprocal accumulation of 2-LTR circles. Since a role of LEDGF/p75 in 2-LTR circles formation was excluded [15], this observation raised the possibility that unintegrated viral cDNA molecules are more intensively degraded in the absence of LEDGF/p75. Thus, this mechanism could prevent a more substantial accumulation of 2-LTR circles in LEDGF/p75-deficient cells. To evaluate this possibility, we analyzed the sequence of 2-LTR junctions isolated from LEDGF/p75-deficient and control cells.

### 3.2. LEDGF/p75 Deficiency Increases the Frequency of Deletions at HIV-1 LTR Termini

We analyzed a total of 139 and 105 unique 2-LTR junctions isolated from LEDGF/p75-deficient and control cells, respectively. These junctions were amplified from a cellular DNA pool obtained from 4 independent HIVluc infection experiments. Representative sequences of the 2-LTR sequences analyzed are shown in Table 1. Intact wild type 2-LTR circle junctions (Table 1, sample HL3a38) were defined by the presence of the sequence GTAC generated by the fusion of the invariant AC and GT dinucleotides located at the 5′ and 3′ ends of the HIV-1 cDNA, respectively. In our analyses, junctions of 2-LTR circles lacking nucleotides (nts) in the 5′ and/or 3′ ends of the HIV-1 cDNA were classified as deletions (Table 1, sample HL3a10) and further grouped as small (<10 nts) or large (>10 nts) deletions.

**Figure 2:**
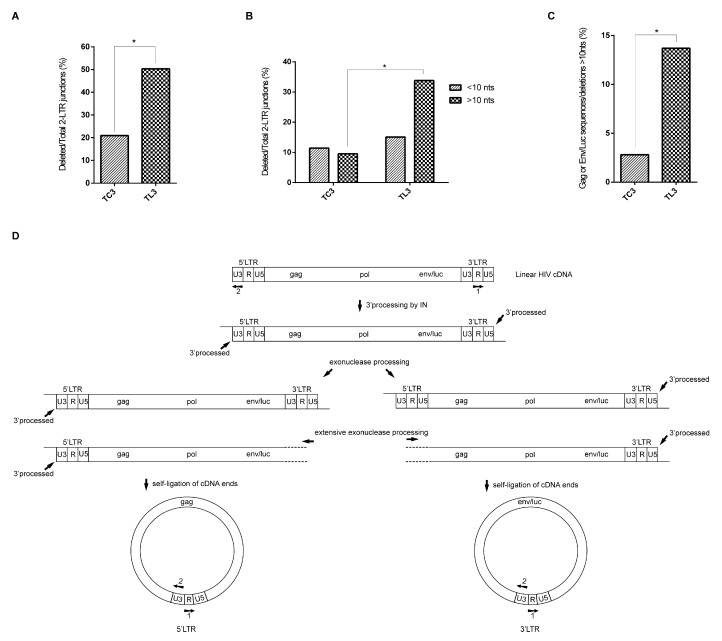
Sequence analyses of deletions at 2-LTR junctions. DNA extracted from 4 independent HIVluc infections of T_C3_ and T_L3_ cells was pooled and used for 2-LTR junctions PCR amplification. (**A**) Overall percentage of 2-LTR junctions displaying deletions; (**B**) data shown in (A) was subdivided into 2 categories to illustrate the frequency of short (<10 nts) versus large (>10 nts) deletions among the analyzed sequences; (**C**) frequency of sequences harboring large deletions (>10 nts) with the inclusion of *gag* or *env*/*luc* gene fragments; (**D**) Schematic representation of the mechanism responsible for generating the sequences shown in (C). “*” indicates significant statistical difference (*Z*-test for proportions).

In our analysis, we observed statistically significant differences (*p* < 10^−4^) in the overall frequency of 2-LTR junction deletions in LEDGF/p75-deficient when compared to control cells. T_L3_ cells exhibited more than 2-fold deletions than T_C3_ cells (50.35% versus 20.9%) (Figure 2A), and large deletions (>10 nts) were 3-fold over represented in LEDGF/p75-deficient than in control cells (*p* < 10^−4^) (Figure 2B). However, the frequency of short deletions (<10 nts) was similar between these cell lines and the disproportion observed in T_L3_ cells between the frequency of small and large deletions was absent in T_C3_ cells. Therefore, these results indicated that large deletions at 2-LTR junctions were enriched in LEDGF/p75-deficient cells.

Surprisingly, we observed a statistically significant (*p* < 3 × 10^−3^) 3-fold increase in the frequency of junctions harboring both large deletions and unusual sequences in LEDGF/p75-depleted cells as compared to the control cells (Figure 2C). A fraction of these junctions contained a non-processed U5 end followed by the PBS, gag and a processed U3 end (Table 1, sample HL3a27), whereas others were formed by a processed U5 end followed by sequences of the *nef* or luciferase genes and a non-processed U3 end (Table 1, sample HL3a5). These junctions could result from self-ligation of extensively degraded linear viral cDNA molecules forming 1-LTR circles that contain a 5′ or a 3′LTR and a fragment of the *gag* or *env/luc* genes, respectively (Figure 2D). Importantly, the primers that we used for cloning the 2-LTR junctions could have amplified the proposed 1-LTR circles (Figure 2D).

An alternative explanation for the genesis of the junctions represented in Figure 2C is autointegration of 3′ processed linear HIV cDNA molecules. However, this mechanism seems unlikely considering the negligible role of LEDGF/p75 in autointegration [22]. Moreover, our experimental conditions did not favor detection of autointegrants reported to peak at 10 h post-infection and to rapidly decrease over time [22]. The HIV-1 cDNA used in our studies was isolated 24 h post-infection, which corresponds to the time point where 2-LTR circles level is the highest in T_L3_ and T_C3_ cells [16].

### 3.3. HIV-1 Reverse Transcription Activity Is Not Affected by LEDGF/p75 Depletion

The elevated frequency of deleted 2-LTR junctions observed in LEDGF/p75-deficient cells could result from altered HIV-1 reverse transcriptase (RT) activity that fails to produce full-length HIV-1 cDNA products or to generate the correct HIV-1 cDNA termini. Therefore, we evaluated this potential mechanism.

A detrimental effect of LEDGF/p75 deficiency on the processivity of RT can be excluded since HIV-1 cDNA levels are similar in cells lacking or not lacking LEDGF/p75 (Figure 1B and references [16,17]). Then, we defined the effect of LEDGF/p75 on the fidelity and processivity of the RNase H activity of RT that generates the 3′ and 5′ viral cDNA ends [23]. RT-mediated cDNA synthesis (minus-strand DNA) initiates from the tRNA primer using as template the positive single-stranded viral RNA. This tRNA primer is later removed, except for the last ribonucleotide, by the RNase H defining the right (U5) viral cDNA end [24,25,26,27]. In contrast, the left end of the linear viral cDNA (U3) is defined by an independent molecular mechanism that requires removal of the polypurine tract (PPT) primer implicated in a second event of RT-mediated cDNA synthesis (plus-strand DNA) by RNase H [23,28]. Therefore, errors in the RNase H-mediated removal of nucleotides downstream of the U3/PPT or U5/tRNA normal boundaries or the DNA strand transfer events lead to U5 or U3 biased abnormal HIV-1 cDNA termini [13,29,30,31].

**Figure 3:**
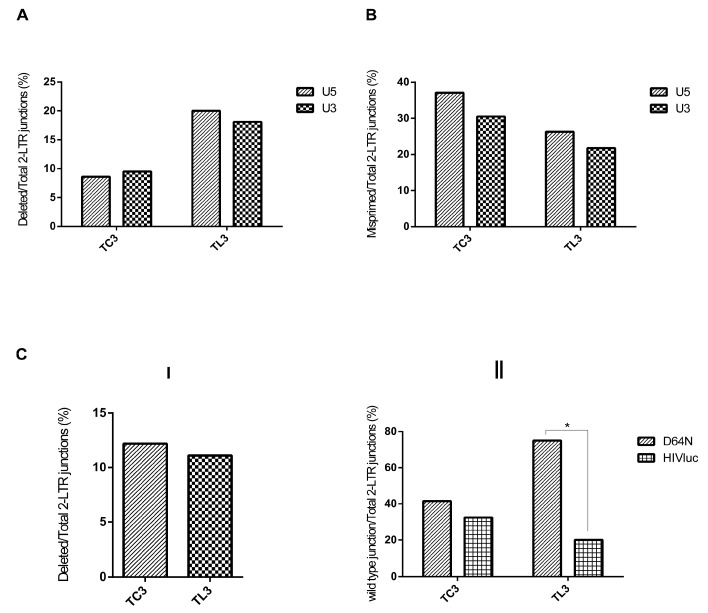
Quantification of 2-LTR junctions generated by aberrant reverse transcriptase or integrase activity. (**A**) Frequency of deletions at the U5 and U3 regions of 2-LTR junctions. Data shown in Figure 2A was re-organized to show the incidence of deletions at the U5 and U3 regions; (**B**) frequency of 2-LTR junctions inferred to arise from reverse transcriptase mispriming events; (**C-I**) frequency of 2-LTR junctions displaying deletions in cells infected with HIV-1_D64N_; (**C-II**) frequency of wild type junctions in cells infected with HIV-1_D64N_ or HIVluc.

Analysis of deleted 2-LTR junctions (Figure 3A) indicated similar frequencies in U5 (8.65%) and U3 (9.5%) in T_C3_ cells that did not account for statistically significant differences (*p* = 0.82). In addition, deletions at U5 and U3 were similar in T_L3_ and T_C3_ cells (20% and 18.1%, *p* = 0.69). These results indicate that deletions at 2-LTR junctions were not biased to U5 or U3, suggesting that their occurrence was not due to aberrant RNase H activity.

Furthermore, we determined the effect of LEDGF/p75-deficiency on the RNase H activity of RT. Impaired RNase H fidelity generates 2-LTR junctions missing only 1 of the invariant dinucleotides at the U5 or U3 ends due to abnormal removal of deoxynucleotides downstream of the PPT or tRNA primers [29,30]. In addition, defective RNase H activity can result in 2-LTR junctions containing inserted virally-derived sequences [29,30,31,32,33,34] as those observed in 2-LTR junctions derived from both T_C3_ and T_L3_ cells that have PPT or tRNA primer sequences or part of the *env*, *nef* or the luciferase open reading frames plus the PPT sequence (Table 1, samples HL3a23, HL3a26, HL3a42 and HL2A-6). 2-LTR junctions exhibiting these characteristics were grouped as U5 or U3 mispriming.

The frequency of U5 or U3 mispriming 2-LTR junctions was similar in HIVluc-infected T_L3_ and T_C3_ cells (Figure 3B). U5 and U3 mispriming occurred in 26.3% and 22.7%, respectively, of the sequences analyzed from LEDGF/p75-deficient cells (*p* = 0.38). Control cells exhibited 37.1% and 30.4% of U5 and U3 mispriming (*p* = 0.31), respectively. These data indicated that the RNase H activity and fidelity of RT was not altered in LEDGF/p75-deficient cells. Therefore, it is unlikely that the increased frequency of 2LTR junctions harboring deletions could arise from a defective reverse transcription. Furthermore, the differences in the frequency of mispriming between T_C_3 and T_L_3 cells at U5 (*p* = 0.07) or U3 (*p* = 0.12) was not statistically significant. Thus, mispriming occurred at a similar rate regardless cellular LEDGF/p75 abundance. This observation further excludes any role of RT in the genesis of the deleted 2-LTR junctions.

We also evaluated the fidelity of the DNA polymerase activity of RT by quantifying the frequency of mutations (point mutations/number of analyzed nucleotides) at the HIV-1 LTR termini. A total of 62 different 2-LTR junctions (9500 nts) isolated from infected T_L3_ or T_C3_ cells were analyzed. Virtually identical frequencies (6.9 × 10^−3^, T_L3_ and 6.5 × 10^−3^, T_C3_) were observed for both cell lines. These values are within the range of the reported rate of mutations introduced by RT [35].

Taken together, these findings indicated that HIV-1 reverse transcription is not affected by LEDGF/p75 depletion. Therefore, it is unlikely that the high frequency of deletions at the 2-LTR junctions isolated from LEDGF/p75 cells is due to defective reverse transcription.

### 3.4. Integrase-Mediated 3′ Processing Is a Pre-Requisite for the Increased Frequency of Deletions Observed at the 2-LTR Junctions Isolated from LEDGF/p75-Deficient Cells

It has been reported that inhibition of integrase strand transfer produces less 2-LTR circles in LEDGF/p75-deficient than in control cells, despite of causing a similar impairment in HIV-1 cDNA integration in these cells [15]. However, infection of these cells with a catalytically inactive HIV-1 integrase mutant, that lacks both 3′ processing and strand transfer activities, produces more 2-LTR circles in LEDGF/p75-deficient than in control cells [15]. These data suggest that 3′ processing directs unintegrated HIV-1 cDNA to degradation rather circularization in LEDGF/p75-deficient cells.

To evaluate this hypothesis, we analyzed junctions isolated from T_L3_ and T_C3_ cells infected with an HIV-1 virus harboring an integrase catalytically inactive mutant (HIV-1_D64N_) that is defective for both 3′ processing and DNA strand transfer activity. A total of 77 (41 from T_C3_ and 36 from T_L3_ cells) 2-LTR junctions isolated from pooled DNA corresponding to 3 different infection experiments were analyzed.

HIV-1_D64N_ infection resulted in a low frequency of deletions at the 2-LTR junctions in both T_C3_ (12.2%) and T_L3_ (11.1%) cells (Figure 3C-I) as compared to infections with HIVluc (Figure 2A). Importantly, the differences reported in Figure 3C-I were not statistically significant (*p* = 0.88) in contrast to those represented in Figure 2A. Further comparison of data in Figure 3C-I and Figure 2A revealed that deletions at 2-LTR junctions were 4.5 fold more frequent in T_L3_ cells infected with HIVluc than with HIV-1_D64N_ (50.35% vs. 11.1%, *p* < 0.001). However, deletions at 2-LTR junctions were only 1.7 fold higher in cells expressing LEDGF/p75 infected with HIVluc than with HIV-1_D64N_ (20.9% vs. 12.2%, *p* = 0.22). Therefore, these findings suggest that unintegrated, 3′ non-processed linear HIV-1 cDNA is not a preferred substrate for degradation.

To further corroborate the role of 3′ processing in the degradation of unintegrated linear HIV-1 cDNA we evaluated the frequency of junctions resulting from circularization of non-processed 3′ viral ends (wild type junctions). These sequences retain the terminal invariant dinucleotides in both viral cDNA ends (GT at U5 and AC at U3) (sample HL3a38 in Table 1). In our analysis we considered that levels of 2-LTR circles were reported to be higher in LEDGF/p75-deficient than control cells infected with a HIV-1 class I integrase mutant [15]. Therefore, we expected that if in T_L3_ cells the 3′ non-processed, unintegrated linear HIV-1 cDNA is resistant to degradation, wild type sequences will be more abundant in T_L3_ than T_C3_ cells infected with HIV-1_D64N_. Importantly, our analysis indicated statistically significantly (*p* = 0.003) higher levels of wild type 2-LTR junctions in T_L3_ cells than in T_C3_ cells, 41.5% and 75%, respectively (Figure 3C-II), further supporting the role of 3′ processing in degradation of the unintegrated linear HIV-1 cDNA.

To exclude the possibility that wild type junctions accumulate more in T_L3_ than in T_C3_ cells regardless of 3′ processing, we determined their frequency in cells infected with HIVluc. In this analysis, statistically significantly (*p* = 0.027) higher frequency of wild type junctions were found in control (32.4%) than in LEDGF/p75-depleted cells (20%) (Figure 3C-II). These results are expected considering that 3′ unprocessed genomes will not integrate in control cells and therefore will be enriched in the 2-LTR circle population. In addition, they indicate that T_L3_ cells are not more prone than T_C3_ cells to retain wild type junctions. Therefore, these findings are in agreement with the conclusion that 3′ processing is required for degradation of the unintegrated linear HIV-1 cDNA.

Further analysis indicated that T_L3_ cells infected with HIV-1_D64N_ shown statistically significantly (*p* = 10^−4^) higher levels of wild type 2-LTR junctions than T_L3_ cells infected with HIVluc. However, these differences were not significant in T_C3_ cells (*p* = 0.3) (Figure 3C-II). These results indicate that wild type junctions accumulate selectively in T_L3_ cells infected with HIV-1_D64N_ potentially because of their resistance to degradation in these cells.

## 4. Discussion

Upon nuclear import, the linear HIV-1 cDNA is integrated into the host genome by integrase or targeted by the host DNA repair system resulting in degradation or conversion into circular DNA molecules [1]. As a consequence, inhibition of integration by mutations or drugs impairing the catalytic activity of integrase leads to a marked decrease in provirus formation (approximately 8 fold) and to the accumulation of HIV-1 2-LTR circles [9,10,11,12,13,15]. Intriguingly, although HIV-1 cDNA integration is also severely impaired in LEDGF/p75-deficient cells (8–10 fold) this is accompanied by only a marginal increase in the levels of 2-LTR circles (less than 2 fold) [15,16,17,18], even though LEDGF/p75 does not affect 2-LTR circle formation [15]. This discrepancy was observed in human and mouse cells, excluding a cell type specific phenomenon, and suggests that rather than converted into 2-LTR circles, unintegrated viral cDNA is preferentially degraded in the absence of LEDGF/p75. In this report, we provide evidence suggesting a role of LEDGF/p75 in protecting the 3′ processed linear form of the unintegrated HIV-1 cDNA from exonucleolytic degradation.

We performed a meticulous analysis of the sequences spanning 2-LTR junctions isolated from LEDGF/p75-deficient and control cells. These sequences provide valuable information about the nature of the ends of unintegrated linear forms of HIV-1 cDNA before circularization. Our results indicated that in LEDGF/p75-deficient cells the frequency of deletions at the 2-LTR junctions markedly increased as compared to control cells. Particularly, we observed that deletions at 2-LTR junctions isolated from control cells were preferentially shorter than 10 nucleotides, while larger deletions were favored in LEDGF/p5-depleted cells. These findings suggest that in the absence of LEDGF/p75 unintegrated linear HIV cDNA is subjected to extensive exonuclease activity that prevents proper measurement of 2-LTR circles by quantitative PCR assays [15,16,17]. Thus, this mechanism provides a possible explanation as to why impaired HIV-1 integration in LEDGF/p75-deficient cells does not result in a pronounced increase in 2-LTR circles formation.

In support of our model, pharmacological inhibition of integrase DNA strand transfer activity caused a greater elevation of 2-LTR circles in control than in LEDGF/p75-deficient cells [15]; while in cells expressing LEDGF/p75 this intervention did not affect the frequency of deletions at the 2-LTR junctions [11], or it only augmented by 1.5–2 fold the frequency of small (<10 nts) but not of large deletions that actually decreased by 2–3 fold [12].

Importantly, quantification of total HIV-1 cDNA using gag primers did not indicate differences in HIV-1 cDNA levels in HIV-1-infected LEDGF/p75-deficient and -expressing cells [16,17]. This observation suggests that cellular exonucleolytic activity does not result in complete degradation of the unintegrated linear HIV cDNA in LEDGF/p75-deficient cells. Potentially, HIV-1 cDNA circularization could prevent the complete degradation of the unintegrated viral cDNA. In this case, it is expected that HIV-1 cDNA circular forms generated in the absence of LEDGF/p75 would lack viral DNA sequences located toward the genome termini, thus being more defective for viral gene expression than those generated in cells expressing LEDGF/p75. To support this, analysis of cells at 3 days post-infection with a single-round infection reporter virus, when 2-LTRs represent 45% of the viral cDNA [15], indicated lower HIV-1 gene expression levels in LEDGF/p75-deficient than control cells [36]. Therefore, this novel function of LEDGF/p75 in preserving the integrity of unintegrated linear HIV-1 cDNA could impact the different roles of unintegrated viral cDNA in HIV-1 infection, particularly in primary cells [1,37,38].

Our data also indicate that deletions at 2-LTR junctions require 3′ processing of the viral LTR termini. Deletions were more frequent in T_C3_ and T_L3_ cells infected with a wild type than with a class I integrase mutant HIV-1, and these differences were more marked in LEDGF/p75-deficient cells. Similarly, higher levels of 2-LTR circles were reported in cells lacking LEDGF/p75 infected with a class I integrase mutant HIV-1 than with a wild type integrase virus in the presence of a strand transfer inhibitor, despite that integration was equivalently inhibited [15]. 3′ processing has also been shown to decrease the stability of unintegrated linear HIV cDNA in cells expressing LEDGF/p75 [39,40], indicating that this degradation mechanism is not the result of LEDGF/p75 deficiency and operates under normal circumstances. For example, in LEDGF/p75-expressing cells the junctions of 2-LTR circles formed in the presence of integrase inhibitors targeting the LEDGF/p75 binding site in integrase (LEDGINs), that affect both 3′ processing and strand transfer [41], contain significantly less deletions than the circles formed in the presence of strand transfer inhibitors [42]. Potentially HRP-2, shown to share some LEDGF/p75 roles in HIV-1 infection [43,44], could also protect unintegrated 3′ processed linear HIV cDNA from degradation.

Importantly, our analyses also excluded defective reverse transcription as a cause of the deletions observed at 2-LTR junctions in LEDGF/p75-deficient cells. First, the frequency of sequences potentially originated by mispriming events due to impaired RNase H activity or fidelity was similar in LEDGF/p75-deficient and control cells. Second, deletions at U3 or U5 were unbiased in both T_L3_ and T_C3_ cells. Third, LEDGF/p75 deficiency did not alter the levels of HIV-1 gag DNA indicating that RT-mediated DNA strand transfer was not affected. Furthermore, our data and previous reports [14,17] indicate that LEDGF/p75 deficiency did not affect integrase-mediated 3′ processing, a reaction that requires normal RT-generated viral cDNA ends.

Taken together, our findings provide data supporting an additional role for LEDGF/p75 during HIV-1 infection. We propose that LEDGF/p75 protects 3′ processed HIV-1 viral cDNA from cellular nucleolytic degradation.

## Figures and Tables

**Table 1:**
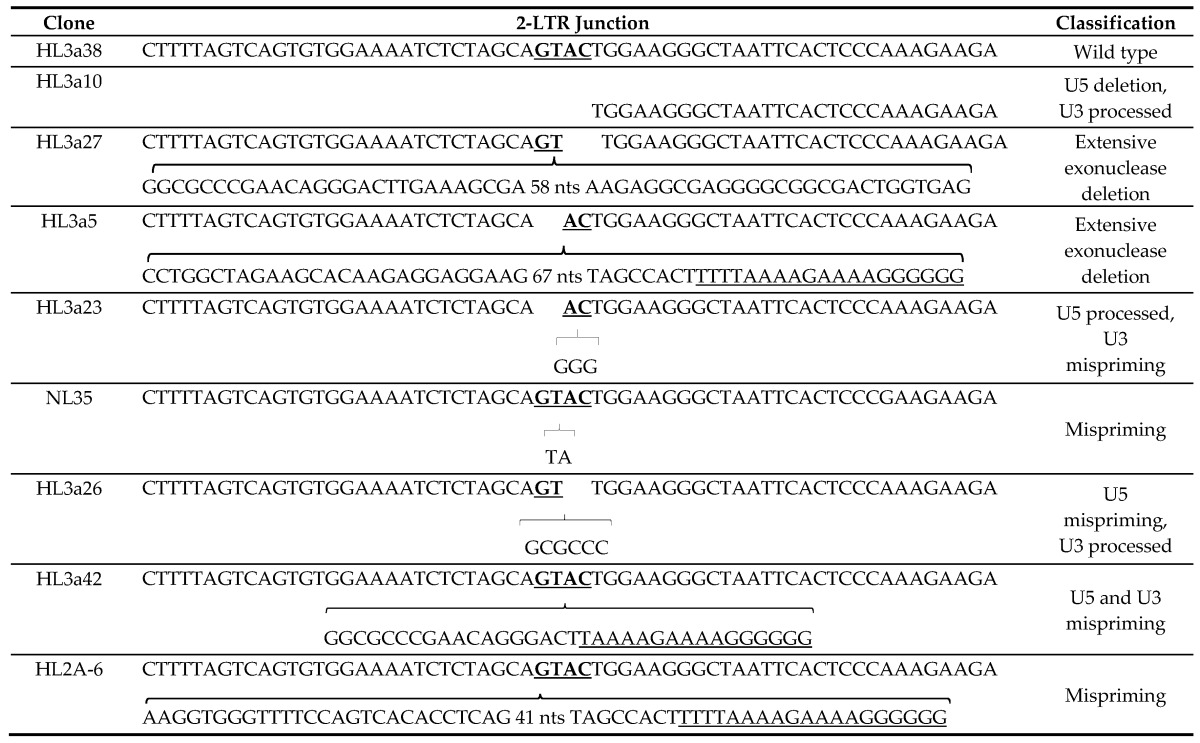
Representative junctions of circularized HIV-1 cDNA containing two long terminal repeats (2-LTRs). HL3a38: unprocessed U5 and U3. Terminal dinucleotides GT (U5) and AC (U3) are in bold and underlined; HL3a10: 50 nts deletion on U5, U3 is processed, an internal nucleotide in U3 is deleted; HL3a27: unprocessed U5 plus insertion of 112 nts corresponding to the *gag* gene, U3 is processed; HL3a5: U5 processed with an insertion of 102 nts of *nef* and U3 unprocessed; HL3a23: U5 processed and 3 nts from the PPT inserted, U3 unprocessed; NL35: U3 and U5 unprocessed plus insertion of a dinucleotide of unspecified origin; HL3a26: U5 unprocessed followed by PBS sequence, U3 processed; HL3a42: U5 and U3 unprocessed with insertions of the PBS and the PPT (underlined) sequences; HL2A-6: U5 unprocessed with an insertion of 41 nts of *nef* and U3 end unprocessed.
